# ER retention receptor, *MoERR1* is required for fungal development and pathogenicity in the rice blast fungus, *Magnaporthe oryzae*

**DOI:** 10.1038/s41598-017-01237-x

**Published:** 2017-04-28

**Authors:** Jaeduk Goh, Junhyun Jeon, Yong-Hwan Lee

**Affiliations:** 1 Department of Agricultural Biotechnology, College of Agriculture and Life Sciences, Seoul National University, Seoul, 08826 Korea; 2 Department of Biotechnology, College of Life and Applied Sciences, Yeungnam University, Gyeongsan, Gyeongbuk 38541 Korea; 3 National Center for Fungal Genetic Resources, Plant Genomics and Breeding Institute, and Research Institute of Agriculture and Life Sciences, Seoul National University, Seoul, 08826 Korea; 4 Fungal Resources Research Division, Nakdonggang National Institute of Biological Resources, Sangju, 37242 Korea

## Abstract

ER retention receptor is a seven trans-membrane protein that plays pivotal roles in function and integrity of endoplasmic reticulum (ER). Insertional mutagenesis of *Magnaporthe oryzae* identified *MoERR1* as a pathogenicity gene encoding putative ER retention receptor orthologous to ERD2 in *Saccharomyces cerevisiae*. Search through the genome identified that *M*. *oryzae* possesses another ortholog of *ERD2*, which is designated as *MoERR2*. When MoERR1 and MoERR2 were tagged with GFP, both were localized to ER. Targeted disruption of *MoERR1* showed pleiotropic effects on phenotypes, while deletion of *MoERR2* had no effect on phenotypes we examined. The disruption mutant of *MoERR1* showed growth retardation and produced significantly reduced number of conidia with aberrant morphology. Appressoria from the mutant were unable to penetrate into plant tissues presumably due to defect in cell wall integrity, thereby rendering the mutant non-pathogenic. The *MoERR1* mutant also appeared to display abnormal ER structure and mis-regulation of genes involved in chaperone function and unfolded protein response under ER stress condition. Taken together, these results suggest that *MoERR1* is a ER retention receptor required for function and integrity of ER, and that *MoERR1*-mediated ER functionalities are essential for fungal development and pathogenesis.

## Introduction

Endoplasmic reticulum (ER) is a membranous cellular structure, which is comprised of interconnected network of tubules, vesicles, and cisternae within eukaryotic cells. ER is known to play roles for post-translational modification, folding and translocation of proteins. These proteins are glycosylated, folded, and/or assembled into multi-subunit complex following their co-translational insertion into the ER lumen or ER membrane before being transported in vesicles to the Golgi apparatus and subsequently further downstream on the secretory pathway^1^. Such ER functionalities are dependent on ER-resident proteins such as chaperons, post-translational modification enzymes, and proteins involved in unfolded protein response (UPR). Retention of these proteins in ER relies mainly on specific retention signals that are present in the primary sequence of the polypeptides and recognized by ER retention receptors. ER retention signal is typically tetra-peptides at C-terminus consisting of (K/R/H/Q/S/A)(D/E/N/Q)EL. In *Saccharomyces cerevisiae* and mammals, HDEL and KDEL are predominant signals, respectively. In *S*. *cerevisiae*, deletion of *ERD2* gene encoding a HDEL receptor was shown to result in failure of BiP protein retention in ER and secretion into extracellular millieu^2^. ER retention receptors are known to localize in ER, Golgi, and intermediate ER-Golgi compartment (ERGIC), and involved in as diverse processes as cell intoxication, ER stress response, and activation of Src family kinases^3^.

*Magnaporthe oryzae* is a causal agent of the rice blast, which is one of the most destructive diseases in cultivated rice^4^. Infection of rice plants by *M*. *oryzae* begins as asexual spores called conidia disseminated and adhere to the leaf surface. Following firm attachment to the substratum, the spore initiates germination in presence of water. Upon recognizing environmental cues such as surface hydrophobicity, germ tube tip differentiates into specialized infection structure called appressorium within 8 hours^5^. Using a penetration peg that translates high turgor pressure generated by appressorium into mechanical force, the fungus breaches the cuticular layer of the host plant and gain access to the tissues. Once inside the plant, it grows invasively with secretion of effectors that contribute to suppression of host immunity and modulation of host metabolism in favor of the pathogen^6^.

In *M*. *oryzae*, a number of studies showed that extracellular proteins such as cutinase, Slp1p and *Avr* proteins play important roles in interaction with host plants during infection^7–10^. Recently, it was shown that two different secretory mechanisms exist for cytoplasmic and apoplastic effectors, of which the latter follow the conventional secretory pathway involving ER^11^. Moreover, study of MoLHS1, one of Hsp70 family proteins residing in ER lumen of *M*. *oryzae* suggested that proper processing of secreted proteins, including effectors, by chaperones in the ER is important for fungal pathogenesis^8^.

In an attempt to identify the genes involved in pathogenicity of *M*. *oryzae*, we previously generated large collection of T-DNA mutants. Here we describe the functional analysis of one of pathogenicity genes involved in function and integrity of ER in *M*. *oryzae*. In one T-DNA mutant defective in pathogenicity, T-DNA tagged a gene (ERR1) encoding a putative ER retention receptor. The *MoERR1* was homologus to *ERD2* in *Saccharomyces cerevisiae* ^2^. We found that there is another *ERD2* ortholog (*MoERR2*) in *M*. *oryzae* genome. To elucidate the functions of *MoERR1* and *MoERR2*, here we investigated roles of the genes in function and integrity of ER through targeted gene disruption/deletion and assessed the contribution of ER functionalties to fungal development and pathogenesis. Our study demonstrates the importance of *MoERR1-*mediated function and integrity of ER in fungal pathogenesis.

## Results

### Identification of *MoERR1* and *MoERR2* encoding ER retention receptor

**Figure 1:**
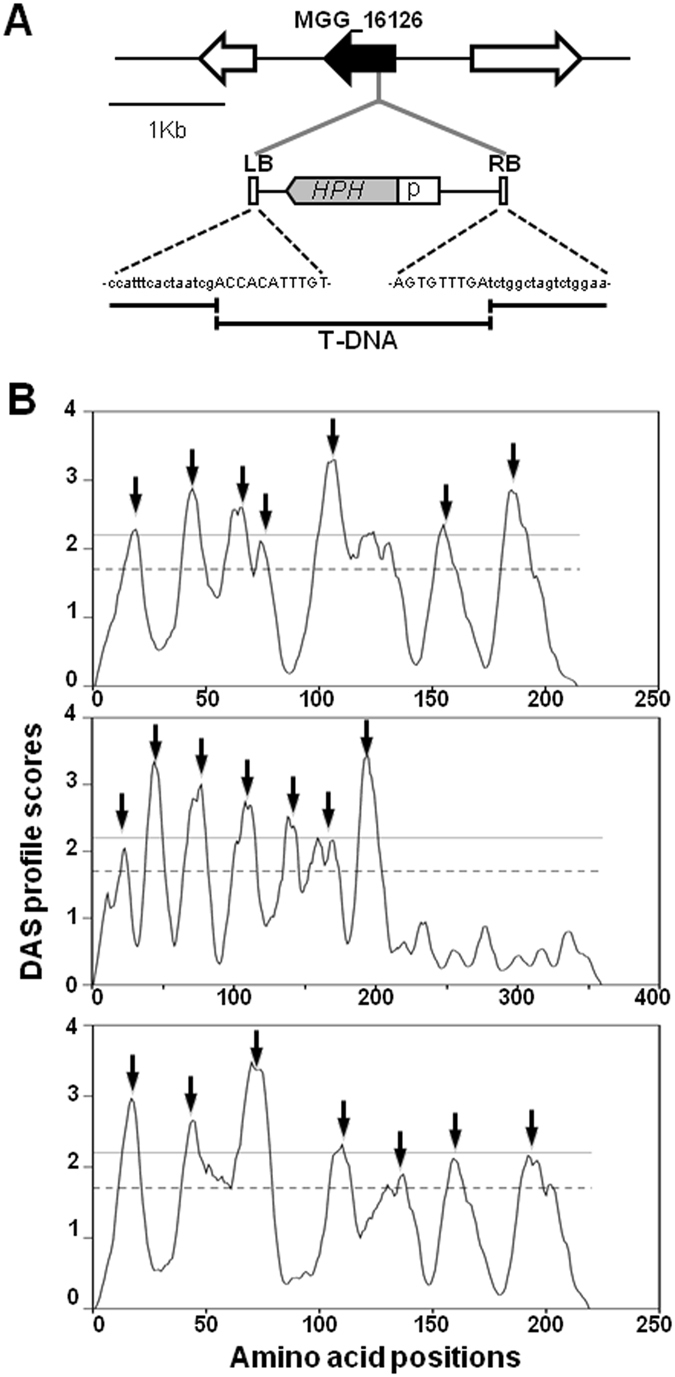
Identification of T-DNA insertion in *MoERR1* ^*T-DNA*^ and hydropathy plot for MoERR1, MoERR2, and ERD2. (**A**) DNA sequences of genome in and around T-DNA insertions sites. Upper cases represent left and right border sequences of T-DNA and lower cases genomic sequences. (**B**) Hydropathy plot MoERR1, MoERR2, and ERD2 (from top to bottom). Transmembrane regions were predicted by DAS server (http://www.sbc.su.se/~miklos/DAS/). Solid and dotted lines indicate strict (2.2) and loose threshold score (1.7), respectively. Arrows indicate transmembrane regions predicted with loose threshold.

ATMT0659D4 (*MoERR1* ^*T-DNA*^) was identified through high-throughput screening of mutant library generated by *Agrobacterium tumefaciens*-mediated transformation as a pathogenicity-defective transformant^12^. Southern hybridization using *HPH* cassette probe revealed insertion of a single copy of T-DNA in the genome of *MoERR1* ^*T-DNA*^. Thermal asymmetric interlaced PCR (TAIL-PCR) revealed T-DNA insertion into MGG_16126 with deletion of 2 bps in genome (Fig. 1A). T-DNA was inserted 370 bp away from the start codon, which corresponds to the third intron of the gene. As translation product of MGG_16126 locus showed highly homology to a yeast ERD2 encoding ER membrane protein (54% identity), we designated MGG_16126 as *MoERR1* (*Magnaporthe oryzae* ER Retention Receptor 1). In addition, we identified another gene encoding putative ER retention receptor (MGG_03681) in *M*. *oryzae* genome and designated *MoERR2* (37% identity with ERD2). Our survey on representative species across different kingdoms showed that Pezizomycotina fungi including *M*. *oryzae* tend to have two *ERD2* homologs in their genome, while yeast and basidiomycota fungi have one *ERD2* homologue (See Supplementary Fig. S1).

Upon identification of putative ER retention receptors in *M*. *oryzae*, we examined if they are membrane proteins, using hydropathy plot (http://www.sbc.su.se/~miklos/DAS), since ERD2 in *S*. *cerevisiae* is known as a seven trans-membrane protein. Hydropathy plot of ERD2 showed that seven trans-membrane domains could be identified with loose threshold score (1.7), but not with strict threshold (2.2) (Fig. 1B bottom panel). When we applied the same lower threshold to hydropathy score profiles of MoERR1 and MoERR2, seven transmembranes could be defined as well, corroborating that both predicted proteins are likely to be membrane proteins (Fig. 1B top and middle panels).

### Targeted disruption/deletion of *MoERR1* and *MoERR2*

**Figure 2:**
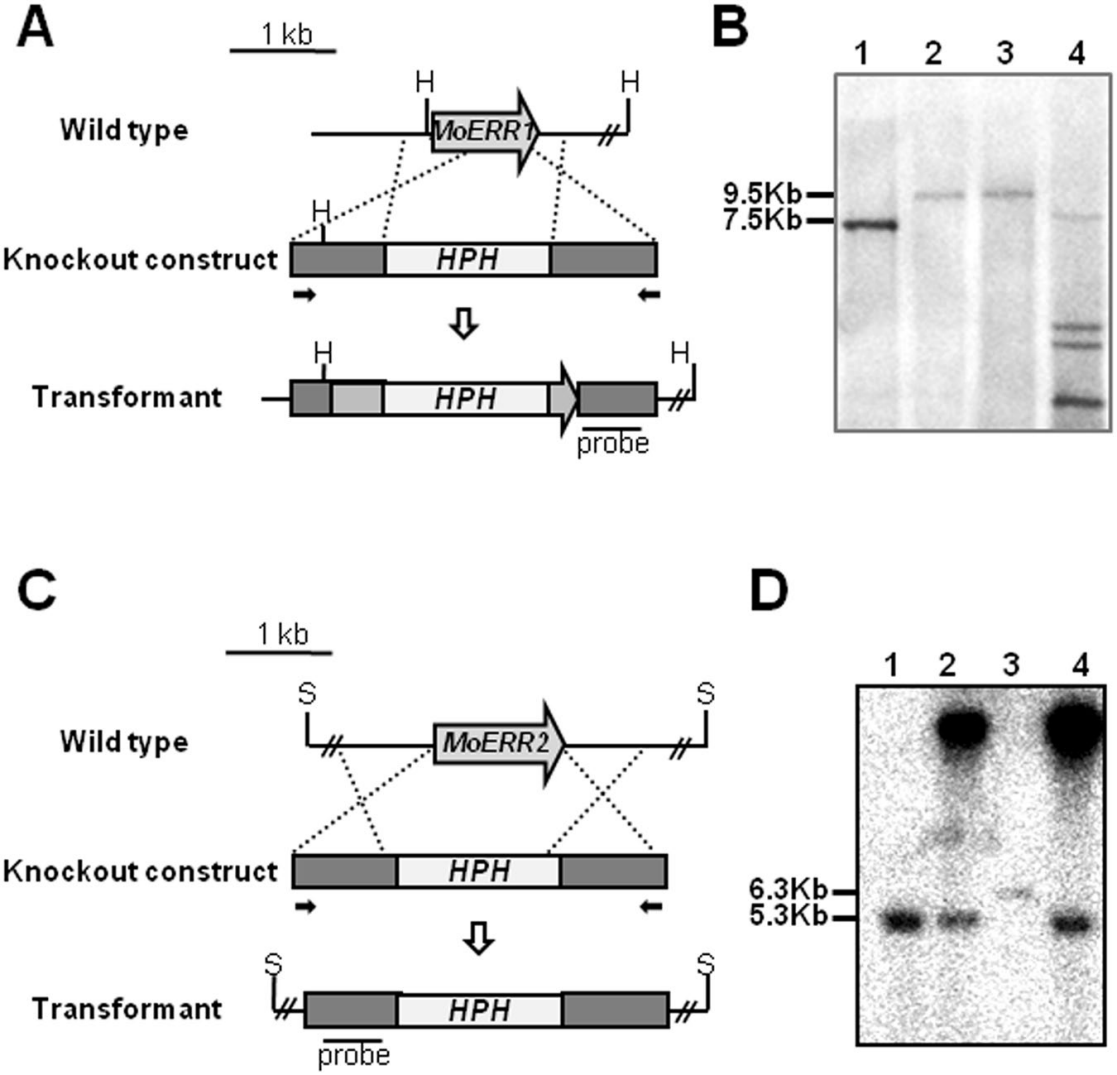
Targeted gene disruption of *MoERR1* and *MoERR2*. (**A**) *MoERR1* gene disruption strategy. *Moerr1* ^*T-DNA*^ was generated by targeted same allele of knock-out construct obtained from *MoERR1* ^*T-DNA*^. (**B**) Southern hybridization of *MoERR1* mutants. Genomic DNA was digested by *Hin*dIII, and probed by 3′ flanking 1 kb fragment. Lane 1: wild type strain KJ201; Lane 2: *MoERR1* ^*T-DNA*^; Lane 3: *Moerr1* ^*T-DNA*^; Lane 4: E1-46 (ectopic transformant). Blot image were cropped for better display. (**C**) *MoERR2* gene deletion strategy using double-joint PCR. Knockout construct was designed to include upstream ~1 kb fragment and downstream ~1 kb fragment of *MoERR2* ORF linked with *HPH* cassette. (**D**) Southern hybridization of *MoERR2* mutants. Genomic DNA was digested by *Sal*I, and probed by 5′ flanking 1 kb fragment. Lane 1: wild type strain KJ201; Lane 2: E1-3 (ectopic transformant); Lane 3: ∆*Moerr2*; Lane 4: E1-46 (ectopic transformant). Blot image was cropped for better display.

In order to investigate functions of MoERR1 and MoERR2 in fungal development and pathogenesis, we attempted to generate gene deletion mutants for individual genes. Using double-joint PCR approach, through which flanking sequences of target gene are fused to hygromycin cassette, we were able to construct knockout vector for *MoERR2* but not for *MoERR1*. To get around this problem, we amplified genomic region encompassing T-DNA insertion sites from *MoERR1* ^*T-DNA*^ and used this fragment containing T-DNA and its flanking sequences (about 1 kb for both sides) as a disruption vector (Fig. 2B). Disruption and knockout vector were individually introduced into wild-type by protoplast transformation (Fig. 2A and C). PCR-based screening and Southern blot analysis for the transformants resulted in six disruption mutant for *MoERR1*, which were designated as *Moerr1* ^*T-DNA*^ and a single deletion mutant for *MoERR2* (∆*Moerr2*) (Fig. 2B and D).

### Subcellular localization of MoERR1 and MoERR2

**Figure 3:**
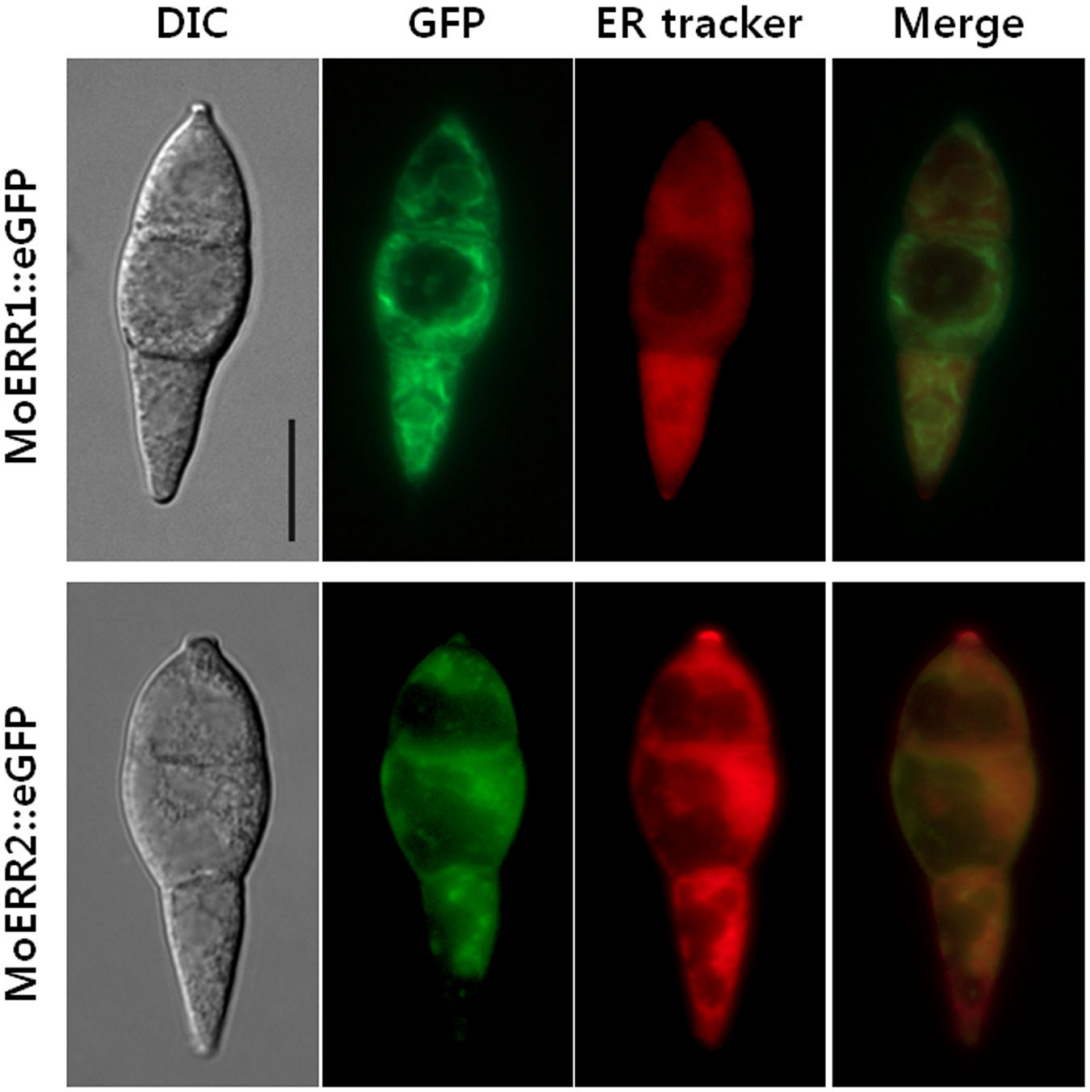
Cellular localization of MoERR1::eGFP and MoERR2::eGFP. MoERR1 or MoERR2 eGFP tagging construct with 1 kb of native promoter region was introduced in wild type strain KJ201. Conidia of GFP tagging strains were stained with 10 mM of the ER-Tracker dye Blue-White DPX, and observed using a 4′,6-diamidino-2-phenylindole filter after incubation for 30 minutes. Blue color of conidia stained by ER tracker was converted to red for better visualization of co-localization. Bar = 10 μm.

Primary sequence similarities and hydropathy plot data prompted us to check localization of MoERR1 and MoERR2. To this end, we produced *MoERR1::eGFP* and *MoERR2::eGFP* under ~1 kb native promoter sequences. The *MoERR1::eGFP* and *MoERR2::eGFP* fragments were cloned into with pII99 vector carrying geneticin resistance gene and introduced into protoplasts prepared from *Moerr1* ^*T-DNA*^ and ∆*Moerr2*, respectively. Using ER-tracker dye, we observed that a high proportion of fluorescence signals from strains expressing either MoERR1::eGFP or MoERR2::eGFP co-localize with signals from ER-tracker staining (Fig. 3). However, MoERR1::eGFP fusion proteins appear to exhibit fluorescent signals around the nucleus and some parts of cytoplasm with a thread-like pattern, while signals from MoERR2::eGFP fusion proteins seem to be distributed without distinct structure. These results, in combination with predicted presence of trans-membrane domains, suggest that both MoERR1 and MoERR2 are membrane proteins that localize to cellular compartments including primarily ER.

### Vegetative growth, conidiation, and conidiophore development

In order to survey effects of *MoERR1* and *MoERR2* during vegetative growth, *Moerr1* ^*T-DNA*^ and ∆*Moerr2* were grown on different conditions including complete medium, minimal medium, carbon starvation and nitrogen starvation media for 9 days post incubation (dpi) (Table 1). For all conditions tested, *Moerr1* ^*T-DNA*^ showed significant difference, compared to the wild type, whereas growth of ∆*Moerr2* was indistinguishable from that of wild-type (See Supplementary Table S4). On complete and nitrogen starvation media, *Moerr1* ^*T-DNA*^ showed 25% reduction in growth, compared to the wild type (Table 1). On carbon starvation and minimal media, *Moerr1* ^*T-DNA*^ showed more severe growth reduction up to 47%, compared to the wild-type.

**Table 1:** Characterization of developmental characteristics in *M*. *oryzae* wild-type, *MoERR1* ^*T-DNA*^, *Moerr1* ^*T-DNA*^ and *MoERR1c* strains.

Phenotype	Wild type	*MoERR1* ^*T-DNA*^	*Moerr1* ^*T-DNA*^	*MoERR1c*
Conidial adhesion (%)^a^	91.66 ± 4.22	23.80 ± 8.37	29.05 ± 8.89	74.96 ± 9.74
Conidial length (μm)^b^	30.63 ± 4.74	21.71 ± 6.57	18.48 ± 4.51	30.29 ± 3.81
Conidial width (μm)^c^	10.37 ± 0.94	9.54 ± 1.67	9.06 ± 1.59	10.27 ± 1.07
Complete medium (mm)^d^	42.33 ± 1.53	30.00 ± 0.00	32.67 ± 2.52	47.50 ± 0.71
Carbon-starved medium (mm)^d^	36.67 ± 3.21	20.00 ± 2.65	21.67 ± 32.67	32.67 ± 0.58
Nitrogen-starved medium (mm)^d^	37.67 ± 2.52	28.33 ± 2.52	28.33 ± 1.15	34.00 ± 4.36
Minimal medium (mm)^d^	46.33 ± 3.06	23.00 ± 1.73	24.67 ± 1.15	47.67 ± 1.15

^a^Percentage of conidia attached to hydrophobic surfaces at 2 hpi. Attached conidia were counted under a light microscope. Data are presented as the mean ± SD from three independent experiments of over 100 conidia each.

^b^Average conidia length (μm). Data are presented as the mean ± SD from three independent experiments of over 100 conidia each.

^c^Average conidia width (μm). Data are presented as the mean ± SD from three independent experiments of over 100 conidia each.

^d^Hyphal growth (mm) was measured at 9 dpi. Data are presented as mean ± SD of three independent experiments.

**Figure 4:**
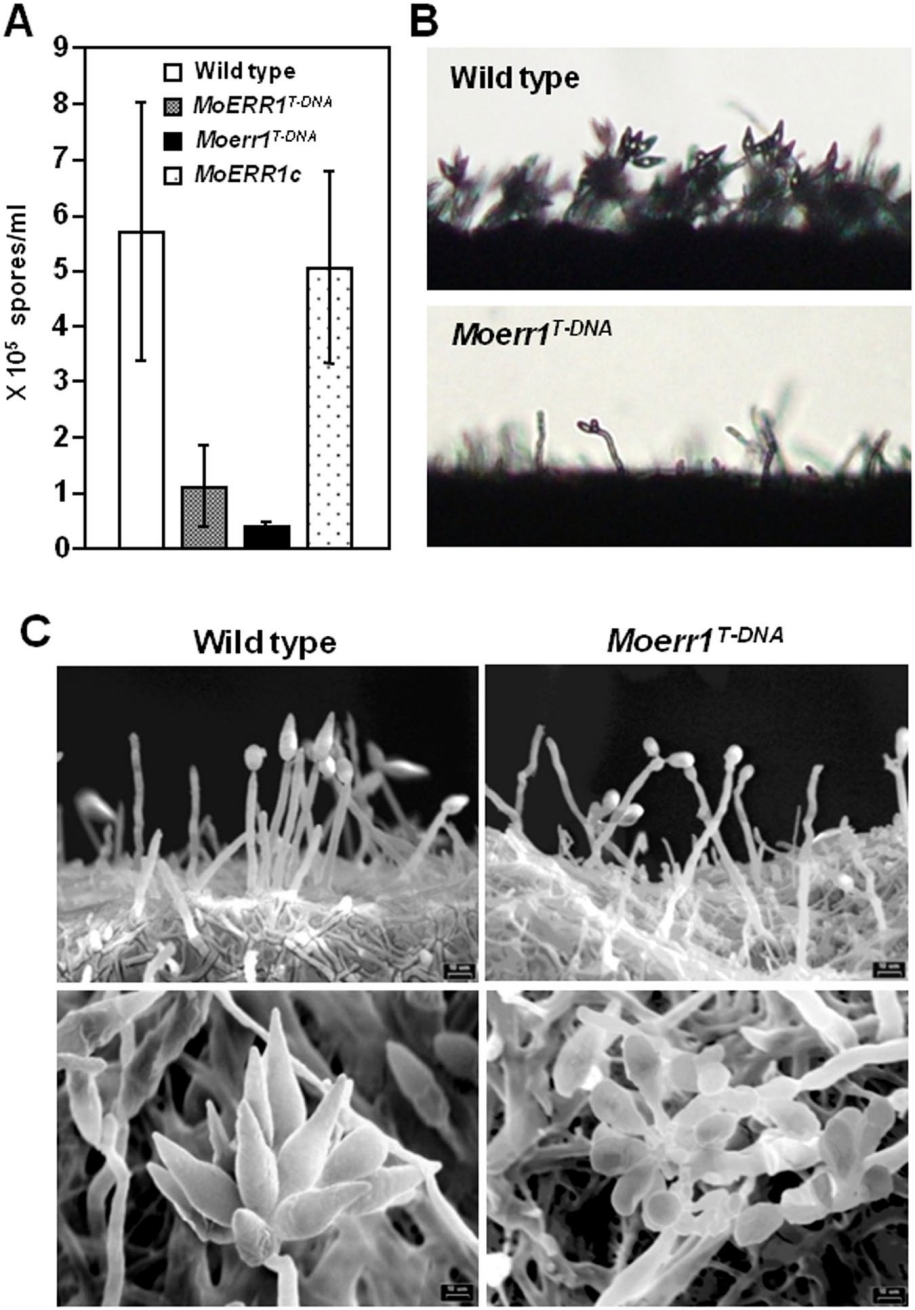
Conidiation and conidiophore development. (**A**) Conidiation was quantified at 10 dpi on oatmeal agar. The values are the means with SD of three replicates. (**B**) Conidiophore development was observed under light microscope at 18 hpi. (**C**) Conidiophore was examined by scanning electron microscopy. Magnification of upper panel was x750 and that of lower panel was x2000.

**Figure 5:**
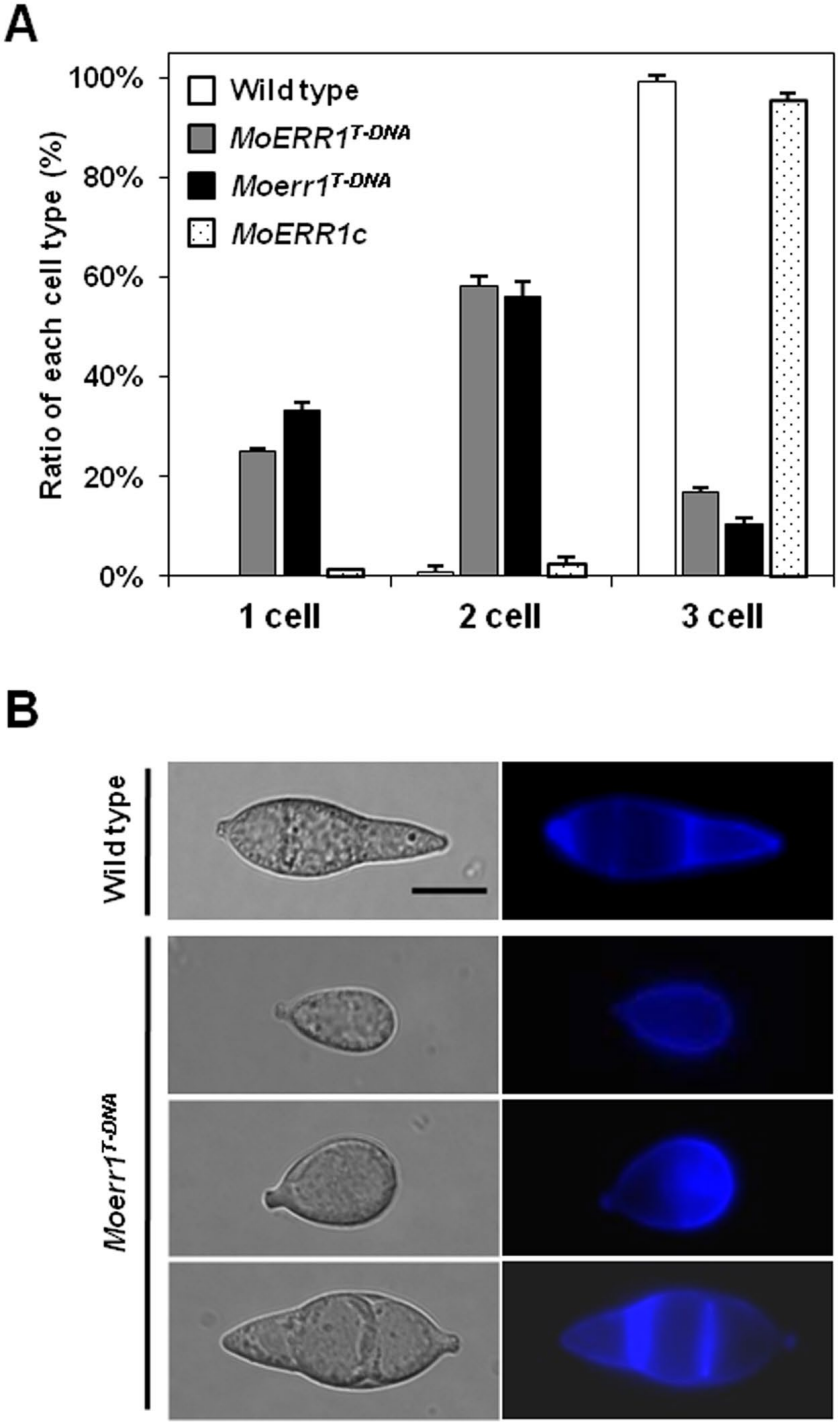
Conidia morphology of *Moerr1* ^*T-DNA*^. (**A**) Distribution of conidia type by cell number. Each ratio of conidia types were calculated with more than 100 conidia examined by 3 replications. (**B**) Cell morphology. Conidial cell wall were stained by calcofluore white, and observed using a 4′,6-diamidino-2-phenylindole filter after incubation for 10 minutes. Bar = 10 μm.

In the *Moerr1* ^*T-DNA*^ mutant, the ability to produce conidia (conidiation) is considerably impaired (Table 1). Conidiation of the *Moerr1* ^*T-DNA*^ was approximately 10% of that of the wild-type (Fig. 4A). However, conidiation and conidia morphology in ∆*Moerr2* was comparable to the wild-type (See Supplementary Table S4). To explain the discrepancy observed in *Moerr1* ^*T-DNA*^, we compared conidiophore development of the wild-type and mutant. The mutant developed conidiophores much less frequently than the wild type at 18 hours post incubation (hpi) (Fig. 4B). Moreover, conidia sporadically developed at the tip of conidiophore in the mutant, in contrast to the conidiophore of the wild-type producing three conidia in a sympodial pattern. Analysis of conidiophore development using Cryo-SEM (Scanned electronic microscopy) confirmed our observation (Fig. 4C). We also observed the differences in number of septa and size in mutant conidia. While the majority (91%) the wild-type conidia consist of three cells reaching 29.6 ± 3.64 μm in length, about 50% of conidia in the mutant was one or two-celled that were measured to be 19.66 ± 4.01 μm in length (Fig. 5A and B and Table 1).

### Germination, appressorium development and pathogenicity

**Figure 6:**
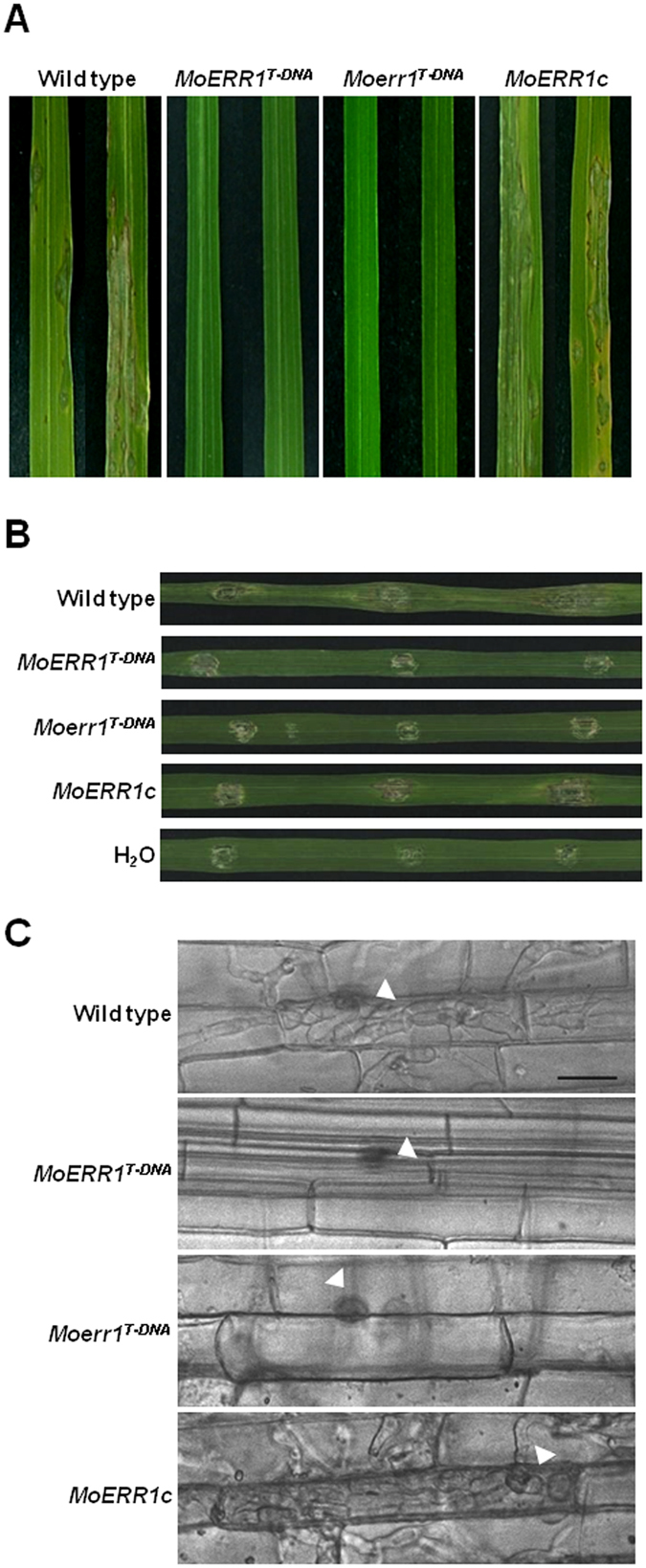
Pathogenicity of wild type and *MoERR1* mutants. (**A**) Spray inoculation. Disease symptoms on rice leaves were examined at 7 dpi on susceptible rice cv. Nakdong. (**B**) Infiltration inoculation. Disease symptoms were examined at 7 dpi on artificial wound. (**C**) Rice sheath infection at 48 hpi. Fungal invasive growth in rice sheath cell was observed under light microscopy. Arrows indicate appressorium. Bar = 20 μm.

To investigate the role of *MoERR1* and *MoERR2* in pathogenesis, conidial suspensions of the mutants were sprayed onto rice seedlings. Our pathogenicity assay showed that *Moerr1* ^*T-DNA*^ is completely nonpathogenic (Fig. 6A), while deletion of *MoERR2* had no effect on pathogenicity (See Supplementary Fig. S2). Even when the *Moerr1* ^*T-DNA*^ was allowed to have direct entry into host tissues via injection of spores through wounded sites, the mutant was unable to develop disease lesions, unlike the wild-type developing large disease lesions (Fig. 6B). Such pathogenicity defect of *Moerr1* ^*T-DNA*^ was recovered to the wild-type level in the complementation strain (*MoERR1c*), demonstrating that not *MoERR2* but *MoERR1* is essential for fungal pathogenicity.

To determine why *Moerr1* ^*T-DNA*^ is not capable of causing disease on its host plants, we examined the infection-related developments in this strain. Conidia produced from *Moerr1* ^*T-DNA*^ did not germinate as efficiently as the wild-type counterparts. They did not germinate at 2 hpi during which majority of wild-type conidia germinate. However, proportion of conidia that germinated and subsequently developed appressoria at 24 hpi showed no difference between the mutant and wild type. Given such delay in germination, we tested if the mutant conidia are able to adhere to the substratum, since tight binding of conidia to underlying surface is known as a prerequisite for conidial germination^13^. Adhesion test using Gelbond film showed that conidia from the *MoERR1* ^*T-DNA*^ and *Moerr1* ^*T-DNA*^ strains are inefficient in attaching to the Gelbond film, compared to the wild-type and *MoERR1c* conidia, suggesting that delay in germination of the mutant conidia is due, at least in part, to defect in adhesion to the surface (Table 1).

Next we asked if appressoria of *Moerr1* ^*T-DNA*^ is functional. When we monitored appressorium-mediated penetration and invasive growth using rice sheath assay, *Moerr1* ^*T-DNA*^ appressoria were incapable of penetration and invasive growth in contrast to wild type forming bulbous invasive hyphae across multiple host cells at 48 hpi (Fig. 6C). These results suggest that defects in pre-penetration development, penetration process, and post-penetration development are all contributing to rendering *Moerr1* ^*T-DNA*^ nonpathogenic. Further investigation of turgor generation within appressorium using cytorrhysis assay indicated that *Moerr1* ^*T-DNA*^ appressoria failed to produce enough turgor pressure for penetration into plant cells to occur (Supplementary Fig. S3A). In presence of high concentration of glycerol, the mutant appressoria underwent more plasmolysis than cytorrhysis, while wild type appressoria predominantly showed cytorrhysis. Indirect measurement of the porosity of the appressorial cell wall using glycerol and polyethylene glycols (PEG) of varying molecular weights^14, 15^, showed that a large proportion of appressoria from the wild type and *MoERR1c* strains underwent cytorrhysis in all molecular weights of PEGs (See Supplementary Fig. S3B). However, a majority of the *Moerr1* ^*T-DNA*^ appressoria underwent plasmolysis rather than cytorrhysis under the same treatment. Increased plasmolysis/cytorrhysis ratios in the *Moerr1* ^*T-DNA*^ are indicative of an increased degree of porosity, suggesting that *MoERR1* is involved in maintaining cell wall integrity of appressorium.

### Impact of disruption of *MoERR1* on ER integrity and functions

In *S*. *cerevisiae*, *erd2* mutant was shown to result in secretion of HDEL-tagged proteins^2^. In order to test if the same can be observed in *M*. *oryzae*, we extracted total proteins from culture filtrate and mycelia separately and carried out western blot analysis using antibody raised against MoKAR2 (See Supplementary Fig. S4A). However, in culture filtrate, we were not able to detect any signal, unlike in mycelia where expression of endogenous MoKAR2 proteins is confirmed. Notably, more MoKAR2 proteins were detected in mycelia of *Moerr1* ^*T-DNA*^ than wild-type. We reasoned that endogenous expression of MoKAR2 might not be enough to be detected in culture filtrate after secretion of ER-resident proteins that resulted from faltered ER retention system. Therefore, we turned to expressing either Pro_*LHS1*_-*LHS1SP-GFP*-HEEL or Pro_*TrpC*_-*KAR2SP-GFP*-HDEL in the wild-type and mutant, and performed western blot analysis for proteins extracted from culture filtrate using anti-GFP (See Supplementary Fig. S4B). Since excessive amount of proteins were used to ensure detection of secreted proteins, we were able to clearly see the signals in all the samples derived from strains expressing GFP. In contrast to our expectation, however, it appeared that more HDEL- or HEEL-tagged proteins are secreted from the wild-type than the mutant.

**Figure 7:**
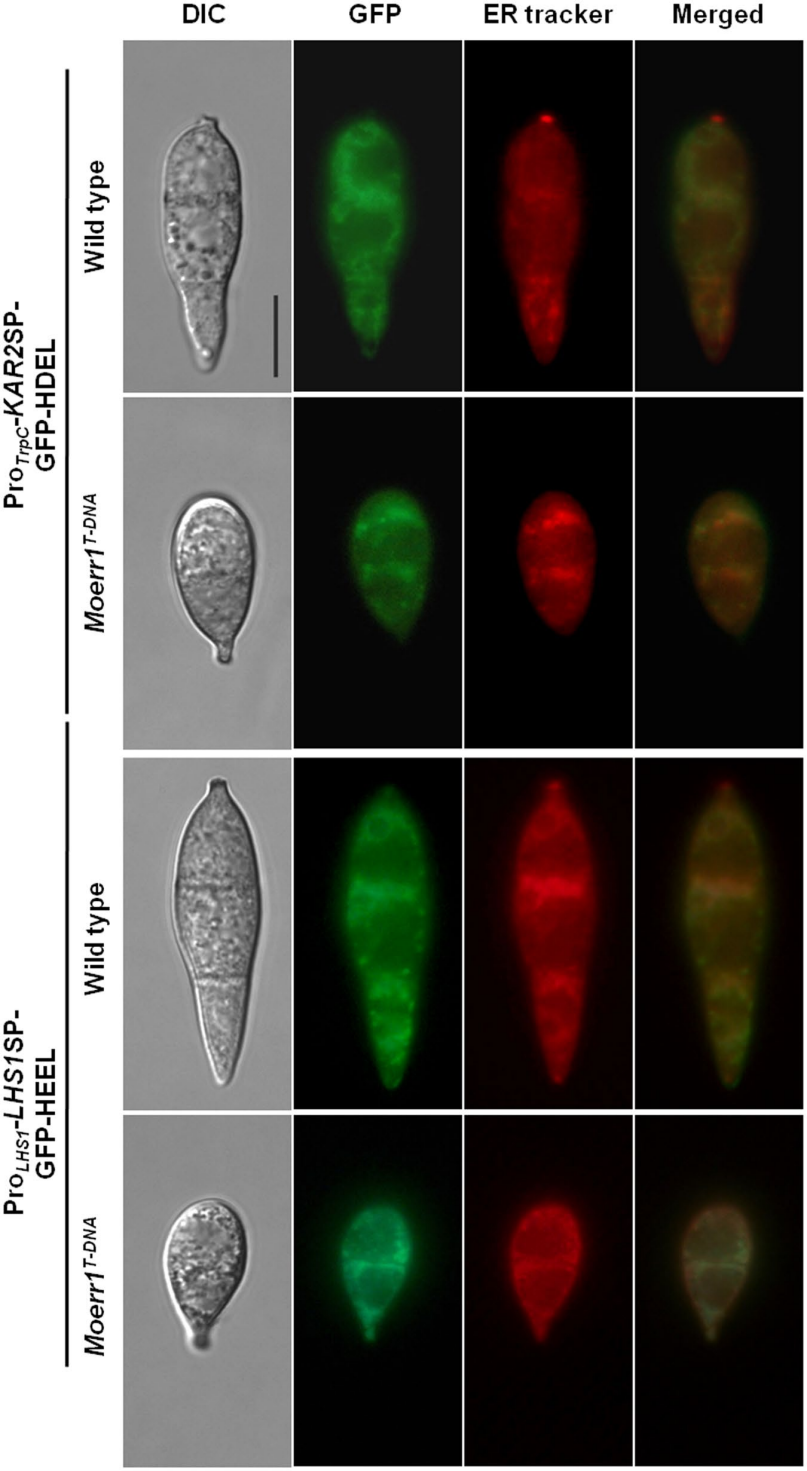
Cellular localization of eGFP-HDEL and eGFP-HEEL in conidia. GFP with ER retention signal construct was designed to have GFP tagging with HDEL/HEEL in N-terminal and signal peptide of LHS1 or KAR2 in C-terminal. Bar = 10 μm.

In the face of this intriguing but puzzling observation, we checked integrity of ER by examining localization of GFP fusion proteins (Fig. 7). Although both fusion proteins carrying signal peptide and ER retention signal at N- and C-terminus, respectively, appeared to co-localize with ER tracker dye in wild type and *Moerr1* ^*T-DNA*^, reticulate membranous network structure was not observed in conidia of *Moerr1* ^*T-DNA*^ unlike that of wild type. Such absence of reticulate ER structure suggests that *MoERR1* is required for maintaining ER integrity.

**Figure 8:**
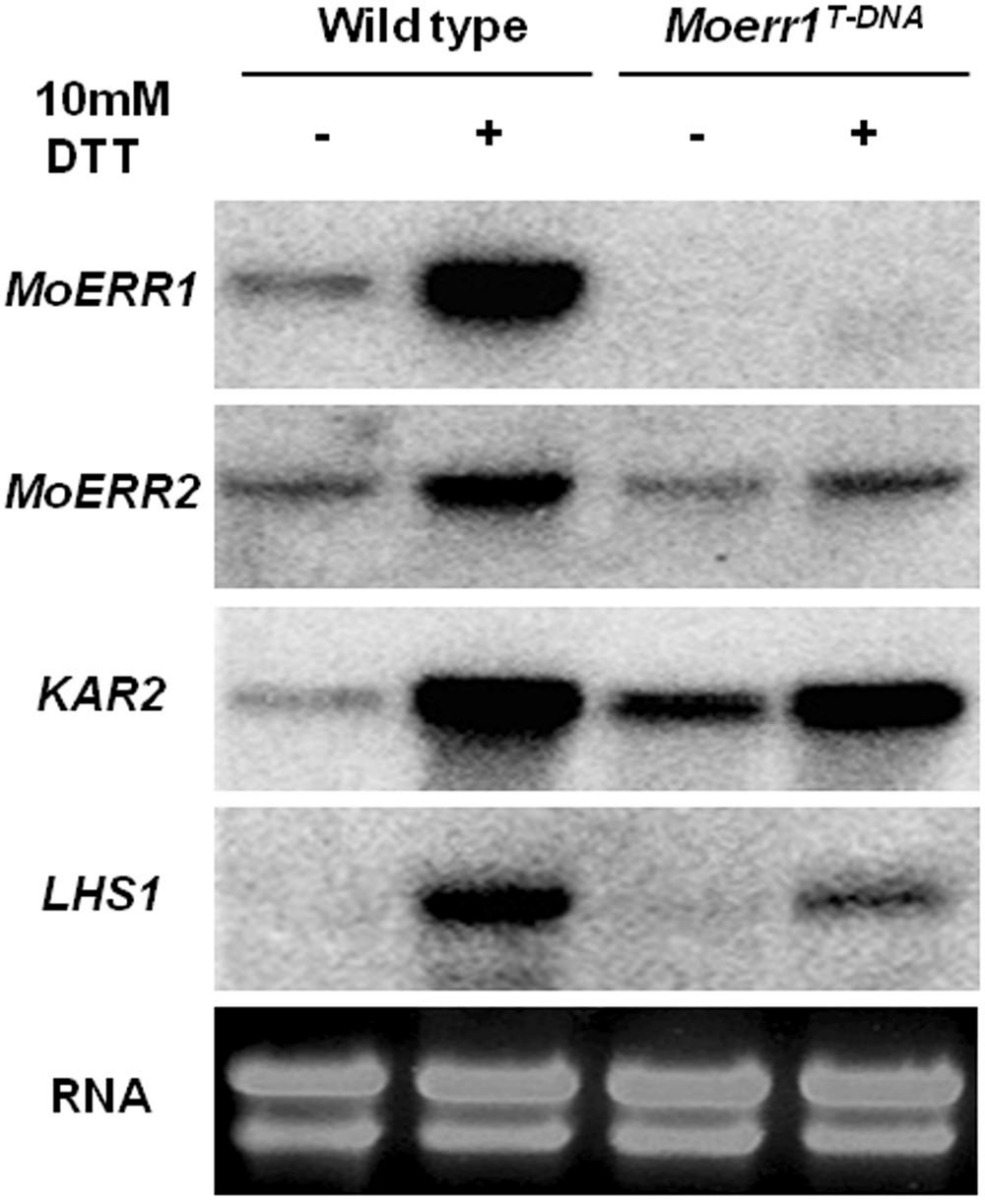
Gene expression of *MoERR1*, *MoERR2*, *KAR2* and *LHS1* under ER stress condition. Mycelia were grown on liquid complete medium for 3 days, and transferred to new medium. After incubation of 1 day, 10 mM DTT treatment for 30 minutes was used as ER stress condition. Partial cDNA fragment of *MoERR1*, *MoERR2*, *KAR2* and *LHS1* were used as probes of northern blot analysis. Blot and gel images were cropped for better display.

Next, we checked what impact disruption of *MoERR1* can have on ER function by characterizing expression pattern of genes encoding ER-resident proteins, *MoKAR2* and *MoLHS1* under ER stress condition using wild-type and *MoERR1* mutant. Transcriptional induction of these genes is important for unfolded protein response (UPR), similar to the yeast UPR. We also observed expression pattern of *MoERR2* in *Moerr1* ^*T-DNA*^. RNA was extracted from mycelia without any treatment or after treatment with 10 mM DTT (Dithiothreitol: an inhibitor of disulfide bond formation) for 30 minutes to induce ER stress, and was subjected to northern blot. DTT has been shown to induce the transcription of genes associated with posttranslational modification in yeast and several filamentous fungi^16, 17^. Expression of *MoERR1*, *MoERR2*, *MoKAR2*, and *MoLHS1* increased to a great extent in response to ER stress, compared to no treatment in wild type (Fig. 8). Even in the *Moerr1* ^*T-DNA*^, *MoERR2*, *MoKAR2*, and *MoLHS1* showed elevated expression level when the fungus was challenged with DTT. However, transcriptional induction of these genes in the mutant was not as strong as in the wild-type. Moreover, we observed that *MoKAR2* expression increased in *Moerr1* ^*T-DNA*^, compared to the wild-type in the absence of ER stress, which is consistent with our western blot analysis showing more MoKAR2 protein in the mutant than wild-type (See Supplementary Fig. S4A). These observations suggest that impairment of *MoERR1* can alter functions of ER by influencing transcription of genes such as *MoKAR2* and *MoLHS*.

### *In silico* identification of ER retention proteins

To envisage the degree of contribution that *MoERR1* can make to functions of ER, we searched for possible ER targeting proteins containing ER-retention signal in C-terminus^18^. HDEL is well-known ER retention sequences in yeast, and KDEL is representative ER retention sequences in mammalian organisms. To increase the sensitivity of our search, we used the presence of (K/R/H/Q/S/A)(D/E/N/Q)EL signature at C-terminal end as our search criterion for putative ER-resident proteins. Among those having this signature, proteins that are predicted as trans-membrane proteins were excluded from the list by TMHMM 2.0 analysis. This led us to identify 43 ER-resident proteins in the proteome of *M*. *oryzae* (See Supplementary Table S2). For comparative analysis, we repeated above steps to all annotated proteins in *A*. *nidulans* and *S*. *cerevisiae*, leading to identification of 36 and 28 putative ER-resident proteins in *A*. *nidulans* and *S*. *cerevisiae*, respectively (See Supplementary Table S3).

The list of ER retention proteins in *M*. *oryzae* contains characterized chaperons such as *KAR2*, *LHS1*, *MHF18*, *PDI* and *SIL1*. ER-resident proteins in our list, except for hypothetical proteins, can be classified into 4 categories – post-translational modification including protein folding, glycosylation and disulfide bond formation, protein transport/trafficking, ER-associated degradation (ERAD), and metabolic pathway. Moreover, we found that 10 ER-resident proteins are shared by all three species of fungi, and more than half of proteins are different from each other (See Supplementary Fig. S4). This observation suggests that different species of fungi may have different ER functions mediated by distinct set of ER-resident proteins.

## Discussion

In our previous studies, we had employed a forward genetics approach to discover and characterize pathogenicity genes in the rice blast fungus *M*. *oryzae* ^12, 14, 15, 19, 20^. Here, we investigated the functions of two genes (*MoERR1* and *MoERR2*) encoding putative ER retention protein in *M*. *oryzae* during pathogenic development, one of which is tagged by T-DNA insertion (*MoERR1*). In *MoERR1* ^*T-DNA*^, a single T-DNA insertion was found in the third intron of the gene with deletion of 2 bp. In northern blot analysis using a probe that hybridizes to the fourth exon of the gene, no transcripts were detected, suggesting that T-DNA insertion prevented full transcription of *MoERR1* gene. Since ER lumen receptor domain spans the entire protein, it is very likely that such interruption of transcription would consequently block production of functional protein.

Our analysis of sequence homology and phylogeny indicated that MoERR1 and MoERR2 are ER retention receptor closely related to ERD2, which is an integral membrane protein regulating retention of ER-resident proteins in *S*. *cerevisiae* ^2^. The dysfunction of *ERD2* gene was shown to result in failure to retain HDEL-tagged proteins and subsequent secretion of them^2^. ER is a membranous cellular structure in eukaryotes, and consists of interconnected network of tubules, vesicles, and cisternae within cells. Diverse functions of ER such as post-translational modifications, proper protein-folding and trafficking hinge on proteins that reside in ER lumen. Retaining such ER-resident proteins in ER, in turn, is dependent on the specialized yet simple system where membrane-embedded ER retention receptors recognize signal in C-terminus of ER-resident proteins^2^. A study of MoLHS1, a chaperone that resides and functions in ER lumen, illustrated the importance of ER function during host infection^8^. Recently, it was shown that apoplastic effectors are secreted via classical secretory pathway including ER and Golgi apparatus^11^, further emphasizing the need to improve our knowledge on ER functions to better understand pathogenesis in the rice blast fungus.

Here, we have demonstrated that MoERR1-mediated ER functions and integrity play essential roles in fungal development and pathogenesis. We have shown that MoERR1 and MoERR2 localize to ER and other structures in close association with ER. Due to our repeated failure to generate deletion mutant for *MoERR1* gene, we instead generated *Moerr1* ^*T-DNA*^ in which T-DNA insertion was reenacted. The phenotypes of *Moerr1* ^*T-DNA*^ were almost identical to those of *MoERR1* ^*T-DNA*^ and could be complemented by introduction of wild-type copy of gene, demonstrating that T-DNA insertion in *MoERR1* gene is responsible for the phenotypic changes in the mutant. The *Moerr1* ^*T-DNA*^ showed defects on conidial morphology, conidia production and conidial adhesion. Moreover, the mutant was unable to penetrate and grow inside the host plants. Unlike disruption of *MoERR1*, deletion of *MoERR2* resulted in no significant phenotypic changes, compared to the wild type. These results indicate that *MoERR1* plays the important roles for asexual development, appressorium-mediated plant infection, and proliferation within host plants in the rice blast fungus.

ER retention signal is tetrapeptides present at C-terminus consisting of (K/R/H/Q/S/A)(D/E/N/Q)EL^18^. To investigate if MoERR1 is *bona fide* ER retention receptor in *M*. *oryzae*, we initially tested secretion of endogenous MoKAR2 proteins carrying ER retention signal using antibody raised against MoKAR2 (See Supplementary Fig. S4A). Based on previous studies, we hypothesized that if ER retention system is altered, MoKAR2 will be secreted into medium and detected by western blot analysis. However, we were not able to detect the presence of proteins in cell-free culture filtrate. Given the amount of proteins inside fungal cells (mycelia), we speculated that our inability to detect secreted MoKAR2 protein in culture filtrate might be due to amount of proteins that are too low to be detected by our approach. In *S*. *cerevisiae*, proteins that are found in culture filtrate were much smaller in quantity than inside the cells, supporting our speculation^2^. Therefore, we chose to utilize GFP fusion proteins carrying either HDEL or HEEL signal at their C-terminus and repeated western blot analysis using anti-GFP. On the contrary to our hypothesis, more GFP proteins were detected in culture filtrate of wild-type than the mutant (See Supplementary Fig. S4B).

Does this suggest that MoERR1 is negative regulator of ER retention as opposed to its known function in yeast and mammals? Examining localization of HDEL- or HEEL-tagged GFP proteins pointed out that it is probably not the case. Close examination of their localization and altered responses to ER stress (DTT treatment) strongly suggested that in the *MoERR1* mutant, integrity of ER structure is severely compromised and impaired (Fig. 7). Based on such observations, it is highly tempting to conjecture that MoERR1 is necessary not only for retention of ER-resident proteins, but also for maintaining integrity and proper functioning of ER, both of which are likely to be inter-related. Therefore, we speculate that loss of ER integrity and function in the mutant may hinder ER-resident proteins from being secreted even when ER retention receptor is lacking in the cell. In support of this, it was observed that dysfuntion of *ERD2* in yeast leads to accumulation of intracellular membranes, concomitant with the inhibition of secretory protein transport through the Golgi complex^2^. It may be that in yeast, presence of ERD1^21, 22^, another ER protein related to ER retention, can attenuate dysfunction of ER in the absence of ERD2, possibly explaining the higher secretion rate of HDEL-tagged proteins in the mutant, compared to the wild type.

To date, only a few studies have investigated roles of ER in development and host infection of fungal plant pathogens. Among those studies that attempted to elucidate association of ER with fungal pathogenicity, a work on *MoLHS1* is noteworthy^8^, since the deletion of *MoLHS1* resulted in phenotypes that are reminiscent of *Moerr1* ^*T-DNA*^ phenotype. The Δ*Molhs1* mutant was severely impaired not only in asexual development but also in both penetration and biotrophic invasion of susceptible rice. Inability of Δ*Molhs1* to establish infection on host plants could be attributed at least in part to defects in proper processing of secreted proteins including effectors. In our study, we observed that disruption of *MoERR1* is more pleiotropic than deletion of *MoLHS1*. The *Moerr1* ^*T-DNA*^ mutant showed defect in conidial morphology as well as appressorial cell wall integrity, which were not reported in Δ*Molhs1* mutant. Through functional analysis of *MoSec22* and *MoVAM7*, it was revealed that membrane trafficking is essential for conidiogenesis and appressorium formation^23, 24^. It is highly likely that disruption of MoERR1-mediated ER retention system can lead to disruption of ER functions and integrity, which in turn affect such membrane trafficking and recycling processes as well. Furthermore, it is plausible based on our data to conclude that disruption of *MoERR1* in *M*. *oryzae* can lead to failure in spatiotemporal regulation of battery of ER-resident proteins including *MoLHS1*.

Many of ER functions, if not all, are mediated by ER-resident proteins. When we catalogued putative ER retention proteins in *M*. *oryzae*, *A*. *nidulans* and *S*. *cerevisiae* using pattern and location information of sequence signature, we found that in these fungi, over a half of ER-resident proteins are predicted to be dependent on KDEL or HDEL sequence for its retention in ER. Moreover, the list contains known ER-resident proteins such as *MoLHS1*, *MoKAR2*, *MHF18* and *MoSIL1*, and many in the list were predicted to be involved in ER-mediated quality control system or UPR pathway, suggesting conserved, core functions of ER among three fungal species. It is of note that there exist ER-resident proteins, which are not found in other two species. Future studies directed to the genes encoding these proteins may provide new insights into ER functions that are associated with species-specific traits.

## Material and Methods

### Fungal strains and culture conditions

*M*. *oryzae* strain KJ201 (wild-type strain) and ATMT0659D4 (*MoERR1* ^*T-DNA*^) T-DNA mutant were obtained from the *Agrobacterium tumefaciens*-mediated transformation mutant library deposited in the National Center for Fungal Genetic Resources (CFGR; http://cfgr.snu.ac.kr)^12^. All strains used in this study were grown on V8 agar (8% V8 juice (v/v), 1.5% agar (w/v), adjusted with pH 6.0 using 10 N NaOH) or oatmeal agar (5% oatmeal (w/v), 2% agar (w/v)) at 25 °C in the constant light in order to promote conidiation. For growth assay on complete media, minimal media and starvation media were used as described previously^25^.

### Gene disruption of *MoERR1* and *MoERR2*

For deletion of *MoERR1*, we were not able to construct knockout vector using double-joint PCR. Instead, the same T-DNA insertion mutant, which we designated as *Moerr1* ^*T-DNA*^ was reproduced as follows: T-DNA insertion site including each 1 kb flanking of 5′ and 3′ region was amplified from genomic DNAs extracted from original T-DNA mutant (*MoERR1* ^*T-DNA*^: ATMT0659D4), and was introduced into wild type KJ201 protoplast. The resulting transformants were selected on TB3 media supplemented with hygromycin (400 μg/ml), and screened by PCR and Southern blot. We obtained 6 gene-disrupted transformants from 96 transformants. The complementation strain, *MoERR1c*, was generated by co-transformation of p491C carrying *MoERR1* gene including its promoter and pII99 harboring geneticin resistance gene. The complementation strains of *MoERR1* mutant were selected on TB3 media supplemented with geneticin (800 μg/ml), and screened by PCR and examination of phenotypes. The *∆Moerr2* was generated using gene replacement construct by double-joint PCR^26^. About 1 kb flanking regions of *MoERR2* ORF were amplified, and fused to geneticin resistance gene. *MoERR2* knockout construct was introduced to wild type KJ201 protoplast, and selected on TB3 supplemented with geneticin (800 μg/ml), and screened by PCR and Southern blot. We obtained a single *MoERR2* deletion transformant from 192 transformants.

### Nucleic acids manipulation and expression analysis

Fungal genomic DNA was isolated by two different methods depending the purpose of experiments. For the southern DNA hybridization and PCR for probe, genomic DNA was isolated from mycelia according to standard method^27^. For the PCR screening in large scale, genomic DNA was isolated as previously described^28^. Southern DNA hybridization was subsequently performed on selected transformants to ensure absence of ectopic integration events. Southern DNA hybridization was performed as previously described^29^. For Northern hybridization, total RNA was isolated from fresh mycelia cultured by Easy-spin^TM^ RNA extraction kit (iNtRON biotechnology, Seongnam, Korea). Total RNA under ER stress was isolated after treatment of 10 mM DTT for 30 minutes in liquid complete media. 18 μg of RNA was used for northern hybridization. Probe of northern hybridization was amplified by PCR, and primer pair was listed in Supplementary Table S1.

### Computational analysis

Annotated gene sequences of *MoERR1* (MGG_16126) and *MoERR2* (MGG_03681) were obtained from *Magnaporthe* genome database in Broad institute. Homology searches of DNA and protein sequences were performed using BLAST algorithms available at the National Center for Biotechnology Information (NCBI). InterPro Scan was performed in European Bioinformatics Institute (http://www.ebi.ac.uk/interpro/). For the phylogenetic analysis, MEGA5.1 was applied^30^. Hydropathy plot analysis was performed on http://gcat.davidson.edu/DGPB/kd/kyte-doolittle.htm. For the computational prediction of ER retention proteins, first we performed regular expression to search putative ER targeting proteins containing (K/R/H/Q/S/A)(D/E/N/Q)EL signature at C-terminus (0–5 mer) among all annotated proteins of *M*. *oryzae*. Then, TMHMM 2.0 analysis was performed to exclude trans-membrane proteins using CFGP website (www.cfgp.riceblast.snu.ac.kr). Finally, 43 putative ER retention proteins were listed in Supplementary Table S2. ER targeting proteins in *S*. *cerevisiae* and *A*. *nudulans* were predicted in the same way as described above. Putative ER targeting proteins in *A*. *nidulans* and *S*. *cerevisiae* are listed in Supplementary Table S3.

Cellular Localization of MoERR1::eGFP, MoERR2::eGFP in wild type and *Moerr1* ^*T-DNA*^. In order to see cellular localization of MoERR1 and MoERR2, we generated MoERR1::eGFP construct and MoERR2::eGFP including native promoter region by PCR. MoERR1::eGFP was introduced to *Moerr1* ^*T-DNA*^ with pII99 carrying geneticin resistance gene. MoERR2::eGFP was introduced to wild type with pII99. For detection of ER retention signal^8^, GFP tagging construct carrying eGFP-HDEL or carrying eGFP-HEEL was used in co-transformation with pII99 into wild type and *Moerr1* ^*T-DNA*^. Localization of GFP tagging strains were observed under UV microscope after staining with 100 nM of ER tracker Blue-White DPX (Molecular Probes) in culture.

### Penetration assay and pathogenicity assay

Penetration peg and invasive growth from appressorium were observed using rice sheath in a procedure described by Koga *et al*.^31^. A 15 µl of conidial suspension was pipetted into rice sheath, and incubated in a humid chamber at 25 °C. Invasive hyphae was observed at 48 hpi by a light microscopy. Pathogenicity assay was performed by spraying conidial suspension (10 ml, 1 × 10^5^ conidia/ml) onto two weeks old susceptible rice cv. Nakdong. For infiltration infection assay, 150 μl of conidial suspension (5 × 10^4^ conidia/ml) was placed on three spots per leaf of three weeks old rice plants (three leaves). The plants or leaves were examined for infection symptoms at 7 dpi.

### Microscopy assay and turgor measurement

Conidia were harvested from 10 days old cultures on OMA and conidial suspensions were prepared at a density of 5 × 10^4^ conidia per ml using sterilized distilled water. A 20 μl droplets of conidial suspension (5 × 10^4^ conidia/ml) were placed on plastic coverslips (Deckglasser, Mülheim, Germany) and incubated in a humid chamber at 25 °C. Conidia morphology was observed using UV microscopy after staining with calcofluor white (100 mg/ml) for 10 minutes. Adhesion assays were performed using Gelbond film (Cambrex BioScience, Rockland, Maine, USA) as a hydrophobic surface as described previously^19^. Appressorial formation was examined as described previously^32^. Briefly, droplets of 20 μl of conidial suspensions (5 × 10^4^ conidia/ml) were placed on plastic coverslips (Deckglasser, Mülheim, Germany) and incubated in a humid chamber at 25 °C. The percentages of germinated conidia and subsequently formed appressoria were measured at 24 hpi. The fractions of germinated conidia and subsequently formed appressoria were measured at 24 hpi. Appressorial turgor pressure was also estimated by performing cytorrhysis in various osmotic solutions^5, 33^. Mature appressoria at 48 hpi were exposed to glycerol or different molecular weights of polyethylen glycol (PEG) solution, and the percentage of collapsed and plasmolyzed appressoria were assessed under a light microscopy^14, 15^.

### Western blot analysis

Fungal strains were grown in liquid complete medium for 4 days for protein assay. Mycelia and culture filtrate were separated by membrane filter using vacuum compressor. Mycelia proteins were extracted by PRO-PREP™ Protein Extraction Solution (iNtRON Biotechnology, Seongnam, Korea). Culture filtrate were precipitated by acetone (4 times of culture filtrate (v/v)) for 2 hours, washed by absolute ethanol for several times, and eluted by SDS-PAGE sample buffer. Measurement of proteins was followed by Bradford assay. Total proteins (25~30 μg) were separated on an SDS-12% polyacrylamide gel and transferred to nitrocellulose membrane (Bio-Rad Laboratories). The blot was probed with monoclonal anti-Green Fluorescent Protein, N-terminal (Sigma-Aldrich) as primary antibody (1:500) and goat anti-mouse IgA + IgG + IgM (H + L) (Pierce Biotechnology) as secondary antibody (1:20,000). Anti-MoKAR2 was used as primary antibody (1:500) and mouse-IgG (Pierce Biotechnology) as secondary antibody (1:20,000).

## Electronic supplementary material

Supplementary Information
