# Supplementary material for: ER retention receptor, MoERR1 is required for fungal development and pathogenicity in the rice blast fungus, Magnaporthe oryzae
Source: Sci Rep. 2017 Apr 28;7:1259. doi: 10.1038/s41598-017-01237-x (PMC5430845; doi:10.1038/s41598-017-01237-x)
Supplement: Supplementary file 1 — Supplementary Information [file 41598_2017_1237_MOESM1_ESM.pdf]

ER retention receptor, *MoERR1* is required for fungal development and pathogenicity in the rice blast fungus, *Magnaporthe oryzae*

Jaeduk Goh[†1a], Junhyun Jeon[†2] and Yong-Hwan Lee[1,3]

[1]Department of Agricultural Biotechnology, College of Agriculture and Life Sciences, Seoul National University, Seoul 08826, Korea

[2]Department of Biotechnology, College of Life and Applied Sciences, Yeungnam University, Gyeongsan, Gyeongbuk, 38541, Korea

[3]National Center for Fungal Genetic Resources, Plant Genomics and Breeding Institute, and Research Institute of Agriculture and Life Sciences, Seoul National University, Seoul 08826, Korea

† Jaeduk Goh and Junhyun Jeon are co-first authors and contributed equally to this work

[a] Present address : Fungal Resources Research Division, Nakdonggang National Institute of Biological Resources, Sangju 37242, Korea

Correspondence to Yong-Hwan Lee

Phone) +82 2 880 4674

E-mail) yonglee@snu.ac.kr

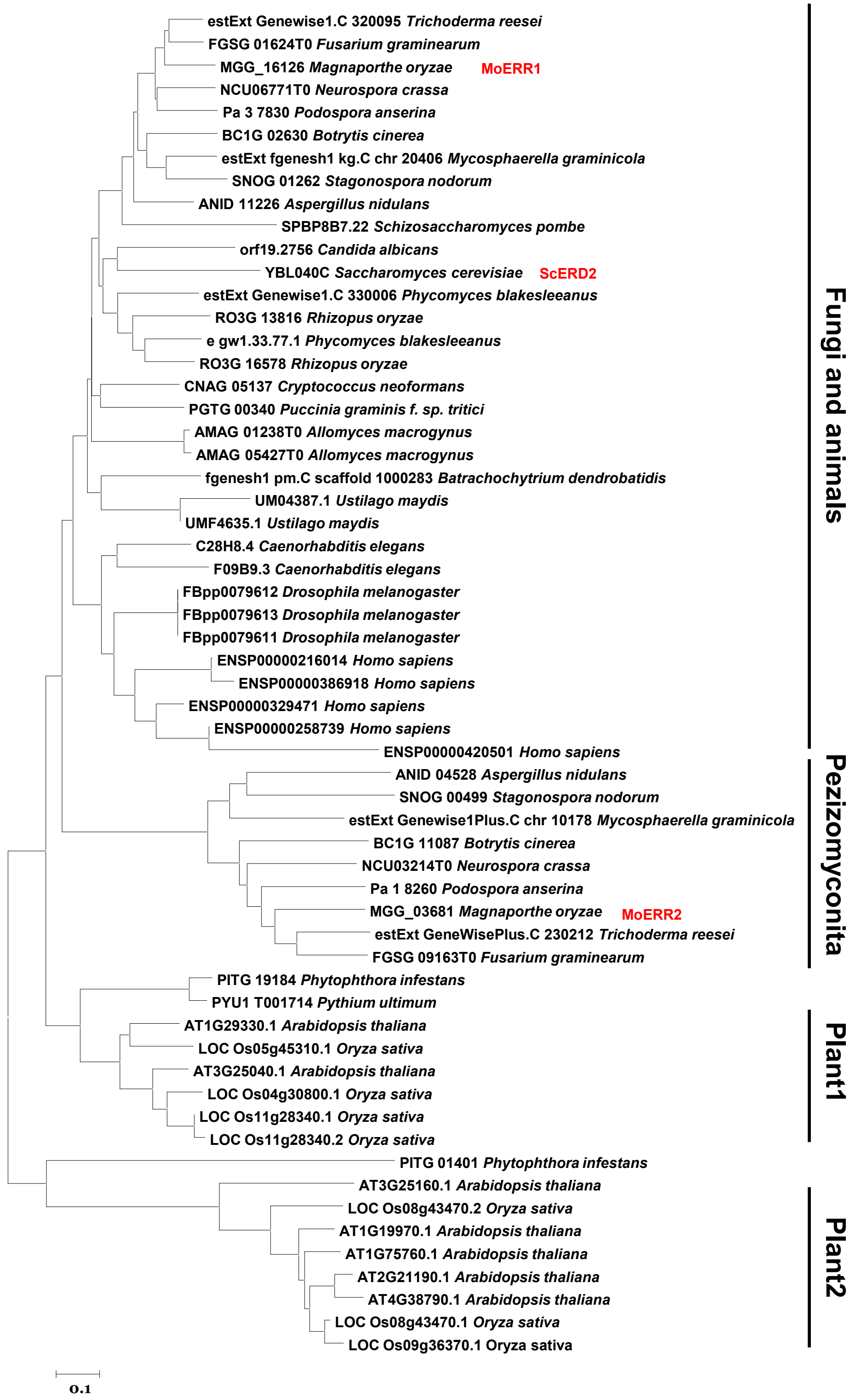

Figure S1. Phylogenetic analysis of MoERR1 and MoERR1 orthologues in other organisms including fungi, nematode, oomycetes, insects, human and plant (http://genomebrowser.snu.ac.kr/). Alignment of collected amino acid sequences and phylogenetic analysis were performed by MEGA 5.1.

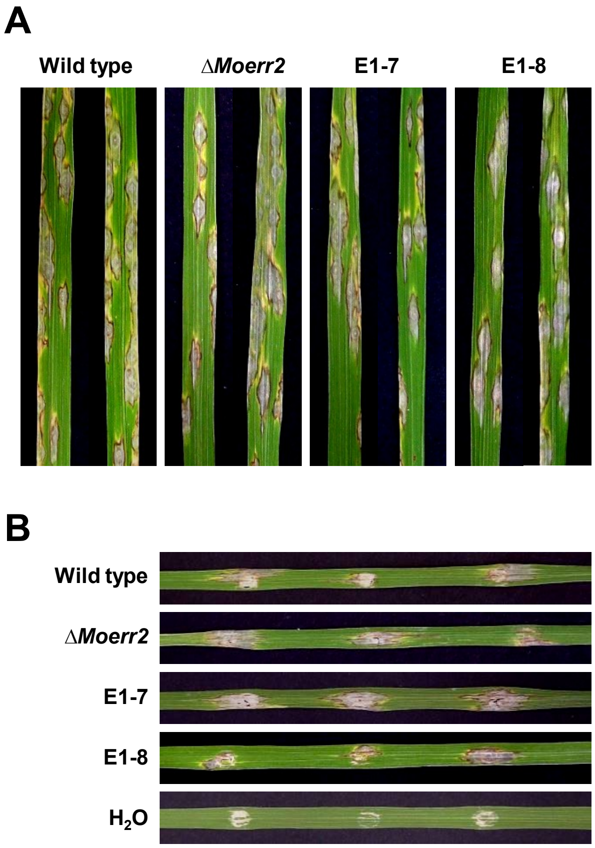

Figure S2. Pathogenicity of *MoERR2* mutant. E1-7 and E1-8 were ecotopic mutants of Δ*MoERR2*.
(A) Spray inoculation. Conidial suspension was 1×10^5 conidia/ml. Conidial suspension were inoculated on 3-week rice cultivar. Pathogenicity were observed at 7 dpi.
(B) Infiltration inoculation. Conidial suspension was 5×10^4 conidia/ml. Conidial suspension were inoculated by artificial wound. Pathogenicity were observed at 7 dpi.

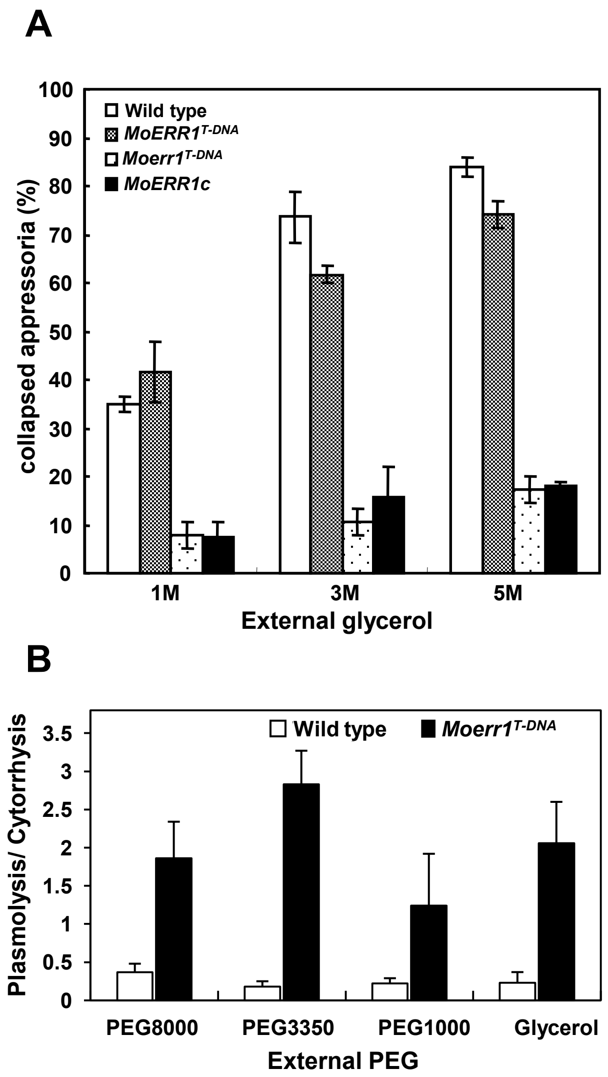

Figure S3. Cytorrhysis assay.

(A) Cytorrhysis assay using glycerol at 48 h.

(B) Ratio of plasmolysis/cytorrhysis treated with PEG8000, PEG3350, PEG1000 and glycerol at 48 h.

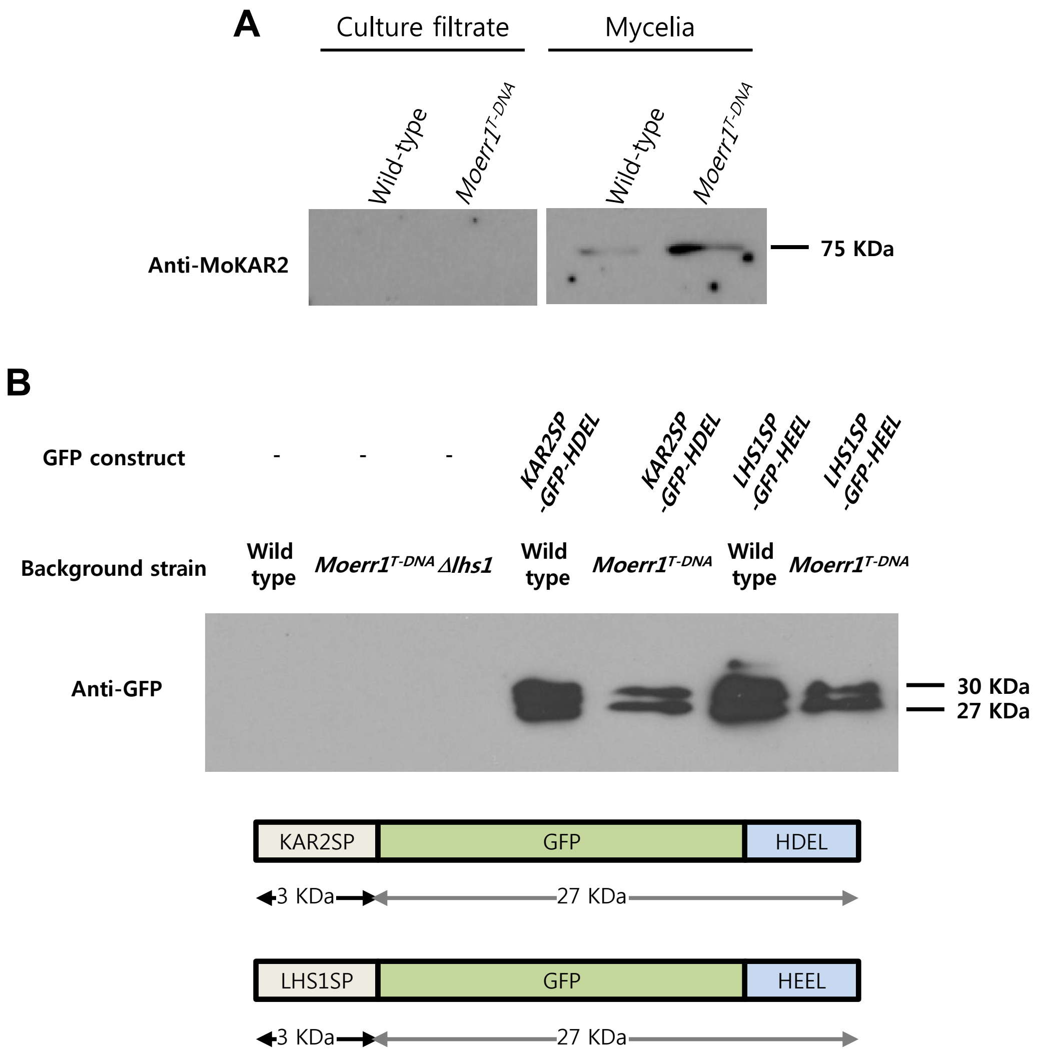

Figure S4. Western blot analysis. (A) Detection of MoKAR2 in culture filtrate. 25ug of total mycelia proteins and culture filtrate proteins were separated in 12% SDS-PAGE gel. Separated proteins were transferred to nitrocellulose membrane and probed with monoclonal anti-MoKAR2. Blot images were cropped for better display. (B) Detection of GFP with ER retention signal in C-terminal and signal peptide in N-terminal. 30ug of total proteins from mycelia were separated in 12% SDS-PAGE gel, and transferred to nitrocellulose membrane and probed with monoclonal anti-GFP. The scheme of the expected protein product and the signal peptide with corresponding molecular mass is depicted described by Yi et al., 2009. Blot image was cropped for better display.

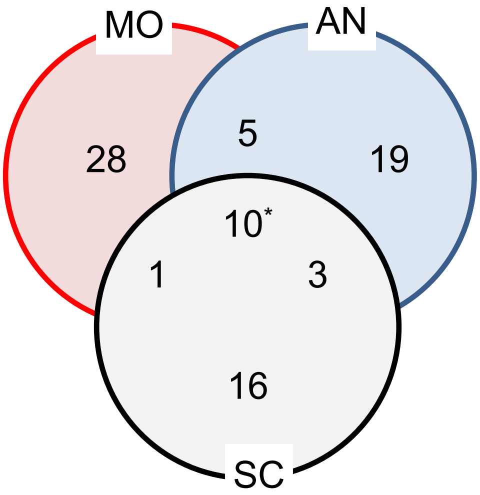

Figure S5. The number of putative genes encoding ER retention proteins shared in *M. oryzae*, *A. nidulans* and *S. cerevisiae*. MO, *M. oryzae*; AN, *A. nidulans*; SC, *S.cerevisiae.* Star mark (*) means different number of genes shared in each organisms because of difference in orthologue numbers. 10 genes in *M. oryzae* are same as 9 genes in *A. nidulans* and 8 genes in *S. cerevisiae*.

Table S1. PCR primers used in this study

Primer name	Sequence (5' → 3')
For generation and confirmation of MoERR1/MoERR2 mutants	
491F	TCAACCCATCATCATTCATTG
491R	ATTTGCCTTAGTGGTTGGTC
Hyg B-F	CGACAGAAGATGATATTGAAGG
Hyg B-R	CTCTAAACAAGTGTACCTGTGC
2423upF	CCAGGTCTGCTTAGCGAG
2423upR	GCACAGGTACACTTGTTTAGAGAACTTGGTATGTCTCGCTGG
3681upF	AGGGCACTTCTTGTCTGATAG
3681upR	GCACAGGTACACTTGTTTAGAGAGATGAAGTTCCGAATGTTGTA
3681downF	CCTTCAATATCATCTTCTGTCGAGACAGCGAAATTGCCTAAG
3681downR	CTTTTTGTTCGTCCGTTCC
For GFP tagging	
MoERR1pF	GTCGTTTTTGTTGGGTTATG
MoERR1gR	CGCCCTTGCTCACCATTACAGGCAGCTTGAACTTTTG

GFP2F	ATGGTGAGCAAGGGC
eGFP/P1	CTTGTACAGCTCGTCCATGCCG
MoERR2gR	CGCCCTTGCTCACCATATCCTTCCACTCATCATTCC
For RNA expression analysis:	
2423RTF	ACGATCCCAATGTTGACAC
2423RTR	GGTACAGTCCCAGAGCAAAC
3681RTF	CTTGCTATCTCCCCATTTGT
3681RTR	ACGTAGAGAGCAACCTGGAC
LHS1qF	ACCCAGTCCTTACCATCAAG
LHS1qR	CTATCTTAACCGGCTTGTCC
KAR2qF	AACGGTCTCGAGAACTATGC
KAR2qR	TCCTTCTGCTCCTCAAAATC

Table S2. *In sillico* identification of ER targeting proteins in *M. oryzae*

locus name	Signal	Characterized gene name	annotation
MGG_01562TO	AEEL		conserved hypothetical protein
MGG_10846TO[**]	AEEL		alpha-galactosidase
MGG_12749TO[*]	AEEL		glutathione reductase
MGG_13360TO	ANEL		conserved hypothetical protein
MGG_02503TO[*]	HDEL	DK1(KAR2)	78 kDa glucose-regulated protein precursor
MGG_03474TO	HDEL		glutaminyl-peptide cyclotransferase
MGG_05753TO[*]	HDEL		disulfide-isomerase
MGG_06786TO[*]	HDEL		disulfide-isomerase A6 precursor
MGG_07502TO[*]	HDEL	MHF18	chaperone protein dnaJ 2
MGG_10843TO	HDEL	SIL1	nucleotide exchange factor SIL1 precursor
MGG_06648TO[*]	HEEL	DK5(LHS1)	hypoxia up-regulated protein 1 precursor
MGG_07234TO	HNEL		FK506-binding protein 2
MGG_09239TO	HQEL		squalene synthetase
MGG_01600TO[*]	KDEL		Sec20 domain-containing protein
MGG_02297TO[*]	KDEL		conserved hypothetical protein
MGG_02811TO	KDEL		acyl-protein thioesterase 1
MGG_03692TO	KDEL		glutaminyl-peptide cyclotransferase
MGG_04045TO	KDEL		mannosyl-oligosaccharide glucosidase
MGG_05242TO	KDEL		conserved hypothetical protein
MGG_05602TO	KDEL		glucosidase 2 subunit beta
MGG_06347TO	KDEL		conserved hypothetical protein
MGG_08164TO[*]	KDEL		disulfide-isomerase erp38
MGG_09082TO	KDEL		conserved hypothetical protein

MGG_09805TO	KDEL	alpha-galactosidase
MGG_05164TO	KEEL	conserved hypothetical protein
MGG_06436TO	KEEL	predicted protein
MGG_11374TO	KEEL	alpha-galactosidase
MGG_13814TO	KEEL	predicted protein
MGG_10480 TO	KQEL	hypothetical protein
MGG_04555TO	QDEL	conserved hypothetical protein
MGG_05688TO*	QDEL	conserved hypothetical protein
MGG_15295TO	QEEL	conserved hypothetical protein
MGG_01375TO	RDEL	conserved hypothetical protein
MGG_02985TO	RDEL	conserved hypothetical protein
MGG_06089TO	RDEL	ribokinase
MGG_11574TO	RDEL	guanidinobutyrase
MGG_04534TO	REEL	chitinase
MGG_10709TO	REEL	phenol 2-monooxygenase
MGG_11443TO	REEL	conserved hypothetical protein
MGG_05389TO **	RQEL	predicted protein
MGG_00503TO	SEEL	diacylglycerol O-acyltransferase 2A
MGG_04364TO	SEEL	conserved hypothetical protein

ER targeting proteins were searched using Regular expression in CROSS-FPP. ER retention signal was [H/K/Q/R/A][D/E/N/Q]EL at extreme C-terminal. Star mark (*) means common ER retention proteins in three fungi including *M. oryzae*, *A. nidulans* and *S. cerevisiae*. Two star mark (**) means species-unique proteins except for *S. cerevisiae*. Two star mark (**) in *S. cerevisiae* indicated Saccharomycetales-specific proteins.

Table S3. Putative ER retention proteins in *A. nidulans* and *S. cerevisiae*.

locus name	Signal	Characterized gene name	annotation
A.nidulans			
ANID_02594	ADEL		conserved hypothetical protein
ANID_06607	ADEL		conserved hypothetical protein
ANID_00932*	AEEL		glutathione reductase
ANID_11853	AEEL		DNA mismatch repair protein
ANID_09413	AQEL		conserved hypothetical protein
ANID_00248*	HDEL		disulfide isomerase
ANID_02062*	HDEL	KAR2	glucose-regulated protein homolog precursor
ANID_06606	HDEL		mannosyl-oligosaccharide glucosidase
ANID_07436*	HDEL		disulfide-isomerase
ANID_10216	HEEL		WD repeat protein
ANID_04467	HNEL		peptidyl-prolyl cis-trans isomerase B
ANID_12271	HQEL		RNA annealing protein Yra1
ANID_00075*	KDEL		disulfide-isomerase tigA
ANID_00221	KDEL		chitinase
ANID_00847*	KDEL		conserved hypothetical protein
ANID_01461*	KDEL		misfolded glycoproteins degradation protein Yos9
ANID_02500	KDEL		putative nicotinamide N-methyltransferase
ANID_04623	KDEL		UDP-glucose:glycoprotein glucosyltransferase

ANID_06170*	KDEL		DnaJ domain-containing protein
ANID_07488	KDEL		agmatinase
ANID_08343	KDEL		FKBP-type peptidyl-prolyl cis-trans isomerase
ANID_10702	KDEL		protein kinase C substrate
ANID_11825	KDEL		conserved hypothetical protein
ANID_07139	KNEL		dienelactone hydrolase
ANID_00868	QDEL		acyl-CoA thioesterase II
ANID_03474	QNEL		conserved hypothetical protein
ANID_10853	QQEL		conserved hypothetical protein
ANID_01510	RDEL		endoplasmic oxidoreductin 1
ANID_02420	RDEL		splicing factor 3B subunit 1
ANID_11694*	RDEL		Sec20 domain-containing protein
ANID_03735	REEL		GTPase activating protein
ANID_05313**	RQEL		conserved hypothetical protein
ANID_06609	SDEL		RING finger domain-containing protein
ANID_00167	SEEL		translation initiation factor eIF-2B alpha subunit
ANID_02522	SNEL		conserved hypothetical protein
ANID_03468	SQEL		histone H2A
S. cerevisiae			
YFR043C**	ADEL	IRC6	Putative protein of unknown function
YPL091W*	AEEL	GLR1	Cytosolic and mitochondrial glutathione oxidoreductase, converts oxidized glutathione to reduced glutathione; mitochondrial but not cytosolic form has a role in resistance to hyperoxia
YOR265W	AQEL	RBL2	Protein involved in microtubule morphogenesis, required for protection from

			excess free beta-tubulin
YCL043C*	HDEL	PDI1	Protein disulfide isomerase, multifunctional protein resident in the endoplasmic reticulum lumen, essential for the formation of disulfide bonds in secretory and cell-surface proteins, unscrambles non-native disulfide bonds
YCR067C	HDEL	SED4	Integral endoplasmic reticulum membrane protein, functions as a positive regulator of Sar1p probably through inhibition of GTPase activation by Sec23p
YDR057W*	HDEL	YOS9	ER quality-control lectin; integral subunit of the HRD ligase
YDR304C	HDEL	CPR5	Peptidyl-prolyl cis-trans isomerase (cyclophilin) of the endoplasmic reticulum, catalyzes the cis-trans isomerization of peptide bonds N-terminal to proline residues
YDR498C*	HDEL	SEC20	Membrane glycoprotein v-SNARE involved in retrograde transport from the Golgi to the ER
YDR518W	HDEL	EUG1	Protein disulfide isomerase of the endoplasmic reticulum lumen, function overlaps with that of Pdi1p; may interact with nascent polypeptides in the ER
YJL034W*	HDEL	KAR2	ATPase involved in protein import into the ER, also acts as a chaperone to mediate protein folding in the ER and may play a role in ER export of soluble proteins; regulates the unfolded protein response via interaction with Ire1p
YKL073W*	HDEL	LHS1	Molecular chaperone of the endoplasmic reticulum lumen, involved in polypeptide translocation and folding; nucleotide exchange factor for the ER lumenal Hsp70 chaperone Kar2p
YOL088C	HDEL	MPD2	Member of the protein disulfide isomerase (PDI) family, exhibits chaperone activity
YOR288C*	HDEL	MPD1	Member of the protein disulfide isomerase (PDI) family
YOR336W	HDEL	KRE5	Protein required for beta-1,6 glucan biosynthesis
YMR214W*	KDEL	SCJ1	One of several homologs of bacterial chaperone DnaJ, located in the ER lumen where it cooperates with Kar2p to mediate maturation of proteins
YKL043W	KNEL	PHD1	Transcriptional activator that enhances pseudohyphal growth
YJL019W	QDEL	MPS3	Nuclear envelope protein required for SPB duplication and nuclear fusion
YNR006W	QEEL	VPS27	Endosomal protein that forms a complex with Hse1p; required for recycling Golgi proteins, forming lumenal membranes and sorting ubiquitinated proteins

			destined for degradation; has Ubiquitin Interaction Motifs which bind ubiquitin (Ubi4p)
YKL057C	RDEL	NUP120	Transcriptional activator that enhances pseudohyphal growth
YLR387C	RDEL	REH1	Cytoplasmic 60S subunit biogenesis factor, associates with pre-60S particles
YOL031C	RDEL	SIL1	Nucleotide exchange factor for the endoplasmic reticulum (ER) lumenal Hsp70 chaperone Kar2p, required for protein translocation into the ER
YJL166W	REEL	QCR8	Subunit 8 of ubiquinol cytochrome-c reductase complex, which is a component of the mitochondrial inner membrane electron transport chain
YDR273W	RQEL	DON1	Meiosis-specific component of the spindle pole body, part of the leading edge protein (LEP) coat, forms a ring-like structure at the leading edge of the prospore membrane during meiosis II
YGL178W	SDEL	MPT5	Member of the Puf family of RNA-binding proteins; binds to mRNAs encoding chromatin modifiers and spindle pole body components
YDR460W	SEEL	TFB3	Subunit of TFIIH and nucleotide excision repair factor 3 complexes, involved in transcription initiation, required for nucleotide excision repair
YBR211C**	SNEL	AME1	Essential kinetochore protein associated with microtubules and spindle pole bodies; component of the kinetochore sub-complex COMA (Ctf19p, Okp1p, Mcm21p, Ame1p)
YBL003C	SQEL	HTA2	Histone H2A, core histone protein required for chromatin assembly and chromosome function
YDR225W	SQEL	HTA1	Histone H2A, core histone protein required for chromatin assembly and chromosome function

ER targeting proteins were searched using Regular expression in CROSS-FPP. ER retention signal was [H/K/Q/R/A][D/E/N/Q]EL at extreme C-terminal. Star mark (*) means common ER retention proteins in three fungi including *M. oryzae*, *A. nidulans* and *S. cerevisiae*. Two star mark (**) means species-unique proteins except for *S. cerevisiae*. Two star mark (**) in *S. cerevisiae* indicated Saccharomycetales-specific proteins.

Table S4. Characterization of developmental charicteristics in *ΔMoERR2*.

Phenotype	Wild type	*ΔMoERR2*
Conidiation(1×10^4 spores/ml)[a]	65.00±16.70	50.00±19.79
Conidial germination (%)[b]	96.82±0.63	98.47±1.41
Appressorium formation (%)[C]	95.85±0.39	96.85±1.08
Conidial length (μm)[d]	30.63±4.74	26.45±4.77
Conidial width (μm)[e]	10.37±0.94	10.36±1.46
Complete medium (mm)[f]	70.00±3.00	70.67±1.15
Carbon-starved medium (mm)[f]	68.67±2.31	71.67±2.08
Nitrogen-starved medium (mm)[f]	56.00±2.65	55.33±1.53
Minimal medium (mm)[f]	73.67±1.15	74.33±0.58

[a] Quantitative conidiation were examined at 10 days on oatmeal agar. Data are presented as the mean±SD from three independent experiments.

[b] Average ratio of conidial germination on cover slip at 8h. Data are presented as the mean±SD from three independent experiments of over 100 conidia each.

[c] Average ratio of appressoium formation on cover slip at 8h (%). Data are presented as the mean±SD from three independent experiments of over 100 conidia each.

[d] Average conidia length (μm). Data are presented as the mean±SD from three independent experiments of over 100 conidia each.

[e] Average conidia width (μm). Data are presented as the mean±SD from three independent experiments of over 100 conidia each.

[f] Hyphal growth (mm) was measured at 12 dpi. Data are presented as mean±SD of three independent experiments.
